# Differentially expressed alternatively spliced genes in Malignant Pleural Mesothelioma identified using massively parallel transcriptome sequencing

**DOI:** 10.1186/1471-2350-10-149

**Published:** 2009-12-31

**Authors:** Lingsheng Dong, Roderick V Jensen, Assunta De Rienzo, Gavin J Gordon, Yanlong Xu, David J Sugarbaker, Raphael Bueno

**Affiliations:** 1 The Thoracic Surgery Oncology Laboratory and the Division of Thoracic Surgery, Brigham and Women's Hospital, Harvard Medical School, Boston, MA, USA; 2 Department of Biological Sciences, Virginia Tech, Blacksburg, VA, USA; 3 Enterprise Research IS and Informatics, Brigham and Women's Hospital and Partners Healthcare Inc, Boston, MA, USA

## Abstract

**Background:**

Analyses of Expressed Sequence Tags (ESTs) databases suggest that most human genes have multiple alternative splice variants. The alternative splicing of pre-mRNA is tightly regulated during development and in different tissue types. Changes in splicing patterns have been described in disease states. Recently, we used whole-transcriptome shotgun pryrosequencing to characterize 4 malignant pleural mesothelioma (MPM) tumors, 1 lung adenocarcinoma and 1 normal lung. We hypothesized that alternative splicing profiles might be detected in the sequencing data for the expressed genes in these samples.

**Methods:**

We developed a software pipeline to map the transcriptome read sequences of the 4 MPM samples and 1 normal lung sample onto known exon junction sequences in the comprehensive AceView database of expressed sequences and to count how many reads map to each junction. 13,274,187 transcriptome reads generated by the Roche/454 sequencing platform for 5 samples were compared with 151,486 exon junctions from the AceView database. The exon junction expression index (EJEI) was calculated for each exon junction in each sample to measure the differential expression of alternative splicing events. Top ten exon junctions with the largest EJEI difference between the 4 mesothelioma and the normal lung sample were then examined for differential expression using Quantitative Real Time PCR (qRT-PCR) in the 5 sequenced samples. Two of the differentially expressed exon junctions (ACTG2.aAug05 and CDK4.aAug05) were further examined with qRT-PCR in additional 18 MPM and 18 normal lung specimens.

**Results:**

We found 70,953 exon junctions covered by at least one sequence read in at least one of the 5 samples. All 10 identified most differentially expressed exon junctions were validated as present by RT-PCR, and 8 were differentially expressed exactly as predicted by the sequence analysis. The differential expression of the AceView exon junctions for the ACTG2 and CDK4 genes were also observed to be statistically significant in an additional 18 MPM and 18 normal lung samples examined using qRT-PCR. The differential expression of these two junctions was shown to successfully classify these mesothelioma and normal lung specimens with high sensitivity (89% and 78%, respectively).

**Conclusion:**

Whole-transcriptome shotgun sequencing, combined with a downstream bioinformatics pipeline, provides powerful tools for the identification of differentially expressed exon junctions resulting from alternative splice variants. The alternatively spliced genes discovered in the study could serve as useful diagnostic markers as well as potential therapeutic targets for MPM.

## Background

Alternative splicing of pre-mRNA is a post-transcriptional process that occurs in approximately 70% or more of all human genes presumably to increase the diversity of the transcriptome and proteome [1]. This process is tightly regulated during development and is different among different tissue types. Inherited and acquired changes in pre-mRNA splicing patterns have been reported in many human malignancies, including colorectal cancer [2], ovarian cancer [3], breast cancer [4], and lung cancer [5,6]. In some cases, specific alternative splicing variants have been proposed as potential clinical markers for cancer diagnosis [7,8] and prognosis [8] as well as function in the role of therapeutic targets [9].

To-date, several groups have reported their efforts to identify novel alternatively spliced genes by analyzing the public available Expressed Sequence Tag (EST) databases [10-12]. Microarray based methods have also been developed to characterize cancer related alternative splicing events using exon arrays and exon junction arrays [13]. Recently, we used whole-transcriptome shotgun pyrosequencing [14] to characterize the transcriptome profiles of individual MPM patient samples and controls obtained at surgery [15]. Approximately 1.6 Gb of sequence data was obtained from ~16 million sequencing reads of ~105 bp average length. We hypothesized that differentially expressed alternative splicing events might be observed in these sequencing datasets.

In the present study, we demonstrate i) that cancer linked alternative splicing profiles can be identified in second generation transcriptome sequencing data *in silico *by mapping all sequence reads onto previously observed exon junction sequences, ii) that this approach can be used to identify expressed exons that are not present in the RefSeq mRNA database at NCBI, and iii) that specific exon junctions can be used to discriminate among MPM and normal samples.

## Methods

### Sequencing data and patient samples

Whole-transcriptome pyrosequencing [14] was previously described for 4 MPM tumors, 1 lung cancer and 1 normal lung using the Roche/454 sequencing system [15]. For this investigation, we used subset of those samples including all 4 MPM samples and one normal sample. Briefly, for each sample, >260 Mb of transcriptome sequence were obtained by shotgun, clonal pyrosequencing using Roch/454 technology. This subset consisted of 13,274,187 total sequencing reads of 105 bp average length. Overall, 98% of the reads matched to human RNA, DNA, and mitochondrial DNA sequences. In each sample, ~15,000 known RefSeq genes were detected by at least one read.

Additional 36 human discarded tissue specimens (n = 18 MPM, n = 18 normal lung) were acquired from our institution's Tumor Bank and used in compliance with a research protocol approved by the Institutional Review Board at Brigham and Women's Hospital.

### AceView transcriptome database

AceView is a public database provided by the National Library of Medicine which provides a comprehensive annotation of transcripts and genes based on data from GeneBank, dbEST and RefSeq. This database was reported to offer a richer view of human transcriptome with 3 to 5 times more high-quality transcript forms than the publicly available databases (e.g., UCSC known genes, RefSeq or Ensemble) [16]. The AceView database of human transcribed sequences ('Aug05') and genome coordinate file ('hg17, May 2004') in GFF format (General Feature Format, is a format for describing genes and other features associated with DNA, RNA and Protein sequences) were obtained from the AceView website [16] and used to define the sequences and locations for 151,486 unique exon junctions.

### Annotation of AceView exon junctions and creation of virtual probes

A customized Active State Perl script was used to index all possible exon junctions for each AceView transcript according to the genomic coordinates of the 3' end of the donor exon and the 5' end of accepter exon that define the exon junction. In Aceview database, there are often multiple transcripts for each gene, so exon junctions with the same (or similar) chromosomal coordinates from different transcript of the same gene will have same mRNA sequence around it. In order to generate non-redundant virtual probe sequences, we eliminated the redundant exon junctions with smaller than 5 bp differences. For each exon junction, a 200 bp transcript segment including 100 bp on each side of the exon junction point was extracted from the longest AceView transcript which contains the exon junction to create a virtual probe sequence for the exon junction. The name of the longest AceView transcript ID containing the junction and exon junction location in the transcript was used as a unique identifier for the exon junction and for the associated virtual probe sequence. For example, the exon junction 'ACTG2.aAug05.574' defines a junction that occurs in the ACTG2 gene in Aceview transcript 'a' in the 'Aug05' database at nucleotide position 574. These probes were also linked to relevant NCBI accession numbers, whenever possible.

### Mapping of 454 transcriptome reads onto virtual probes

**Figure 1:**
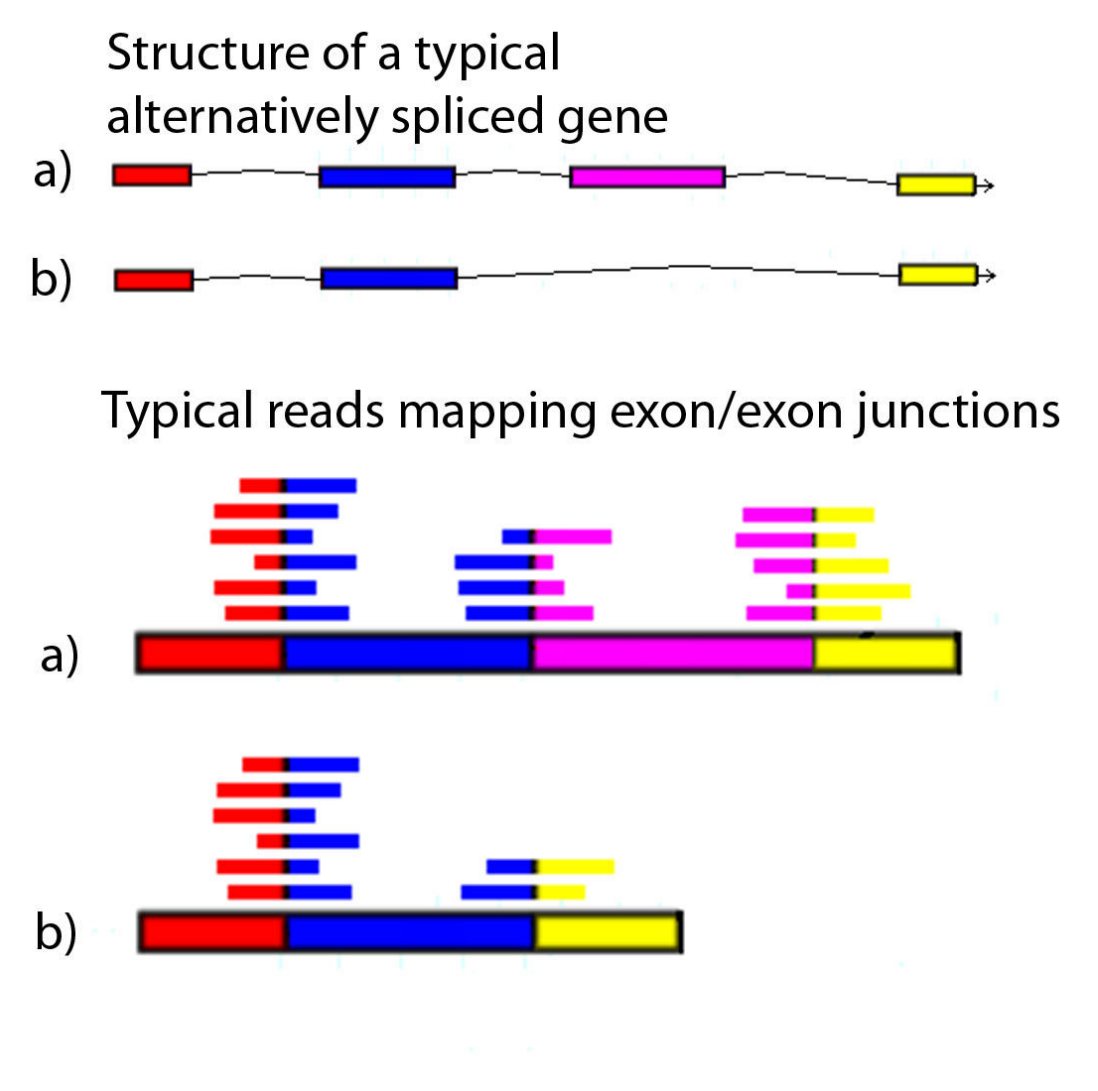
**Identification of alternatively spliced transcripts using second generation sequencing data**. The 454 sequencing reads that span exon junctions provide information about alternatively spliced variants in the human transcriptome. For example, two typical alternatively spliced variants ('a' and 'b') of a single gene are shown in the top half of the figure. The lower half of the figure shows how different 454 reads may align to cDNA sequences generated from the mRNA providing evidence of alternatively splicing events.

The alignment code was run on the Partners Healthcare System High Performance Computing Cluster based on the Microsoft HPC Server 2008 Operating system. NCBI blastall was used to map all the 454 transcriptome reads onto the virtual probe sequences with the following parameters: blastall -p blastn -a 2 -F "m D" -e 1e-10 -m 8. A customized Perl script was used to query the blast results to quantify the number of reads mapping to each virtual probe and cover the junction point with at least 5 bp on both side, at least 35 bp of continuous alignment, and at least 90% identity to the virtual probe sequence. Figure 1 illustrates the theoretical alignment of the ~100 bp long transcriptome reads with the different exon junctions spanned by virtual probes.

### Filtering of mapped exon junctions and generation of an exon junction expression index (EJEI)

To filter out the low-expressed exon junctions across all samples, we required that each unique exon junction map to at least 5 sequencing reads in all patients combined. To filter out the low-expressed genes in any single sample, we also required that at least 4 sequencing reads map to any exon junction in a given gene in each sample. Because there are similar total numbers of reads among the 5 discovery samples [15], additional normalization to the total read count in each sample was unnecessary.

To correct for systematic differences in gene expression levels between the different samples, an exon junction expression index (EJEI) was calculated for each unique exon junction in each sample. The EJEI is the ratio of number of reads that cover a specific exon junction divided by the total number of the reads that map to any of the exon junctions of the gene. This is similar to the Splicing Index reported in ArrayAssist software [17] for analyzing the exon level data on the Affymetrix Exon arrays, however, here we are counting the relative frequency of the exon junctions themselves.

To make the EJEIs comparable among different exon junctions of different genes, they were further normalized by the sum of the EJEI in all patients for the same exon junction. To identify highly expressed exon junctions in MPM relative to normal lung, we generated a ranked list of EJEIs by calculating the differences between the minimum normalized EJEI in the 4 MPM samples and the normalized EJEI for the same exon junction in the normal lung sample. To identify highly expressed exon junctions in normal lung relative to MPM, we similarly generated a ranked list of EJEIs by calculating the difference between the normalized EJEI in the normal lung sample and the maximum normalized EJEI for the same exon junction in the four MPM samples.

### Quantitative Real Time RT-PCR

Total RNA (2 μg) was reverse-transcribed into cDNA using Taq-Man Reverse Transcription reagents (Applied Biosystems, Foster City, CA) and quantified using all recommended controls. Primers (synthesized by Invitrogen Life Technologies) were used at a final concentration of 800 nM in the reaction mixture and are shown in additional file 1. Primer sequences were blasted onto NCBI human Refseq RNA database to confirm that the primers will not amplify other genes. PCR amplification and SYBR-Green-based detection of cDNA was performed using a Stratagene MX3000P machine and default thermal cycling parameters. No-template (i.e., negative) controls that contained water instead of template were run in multiple wells on every reaction plate. An automatically calculated melting point disassociation curve generated after every assay was examined to ensure the presence of a single PCR species and the lack of primer-dimer formation in each well. (The detail method is in Additional file 2).

### Statistical analysis

Average expression levels of exon junctions were compared between MPM and normal samples using a two-tailed student's t-test. Classification accuracy was assessed using a 2 × 2 contingency table and Fisher's exact test. All comparisons were considered statistically significant if *P *< 0.05. All comparisons were conducted using *S-PLUS *software [18]. Post-hoc power analysis was conducted using G*Power [19].

## Results

In total, we indexed 151,486 unique AceView exon junctions to create one virtual probe per unique exon junction. Mapping of the 13,274,187 transcriptome reads against these 151,486 virtual probes revealed 70,953 exon junctions that were covered by at least one sequence read in at least one of the 5 samples using our criteria. The distribution of these 70,953 exon junctions was summarized in Additional file 3.

We used ranked normalized EJEI values to identify the most differentially expressed exon junctions between the four MPM samples and the one normal lung sample. All the exon junctions with EJEI difference bigger than 0.1 were listed examined (see Additional file 4). We identified 76 highly expressed unique exon junctions in MPM and 32 unique highly expressed exon junctions in the normal lung sample (see Additional file 5). We chose to examine in the 5 discovery samples a total of 10 exon junctions: the top 3 ranked exon junctions expressed at relatively higher levels in normal lung and the top 7 ranked exon junctions expressed at relatively higher levels in the MPM samples using real time quantitative RTPCR (see table 1 and additional file 6 for the 10 candidate exon junctions). We arbitrarily weighted our list towards exon junctions over-expressed in MPM tumor because we found more highly expressed exon junctions in MPM (76) than in normal lung (32) and sought to increase our chances of identifying potentially diagnostic alternatively spliced transcripts.

**Table 1:** Normalized EJEI and corresponding RT-PCR results for the top ten differentially expressed exon junctions*

				Normalized EJEI						RT-PCR EJEI result				
						
Normal/Tumor	Gene	Typical AceView sequence	Exon junction location in typical AceView sequence	Meso 1	Meso 2	Meso 3	Meso 4	normal lung	Normalized Meso and Normal EJEI difference	Meso 1	Meso 2	Meso 3	Meso 4	normal lung
normal	EMP2	EMP2.shed3.aAug05	1003	0.000	0.000	0.000	0.000	1.000	1.000	0.079	0.065	0.069	0.071	0.069

normal	CYFIP1	CYFIP1.fAug05	49	0.000	0.000	0.000	0.000	1.000	1.000	0.034	0.056	0.043	0.044	0.052

normal	ACTG2	ACTG2.aAug05	574	0.062	0.000	0.000	0.000	0.938	0.876	0.026	0.054	0.029	0.079	0.209

tumor	C1QA and C1QG	C1QAandC1QG.aAug05	1449	0.221	0.264	0.229	0.286	0.000	0.221	0.139	0.115	0.220	0.190	0.148

tumor	MRPL51	MRPL51.bAug05	542	0.228	0.301	0.215	0.256	0.000	0.215	0.678	0.908	0.710	0.664	0.693

tumor	TXNRD1	TXNRD1.aAug05	1333	0.198	0.220	0.363	0.219	0.000	0.198	0.570	0.507	0.520	0.546	0.461

tumor	CDK4	CDK4.aAug05	1246	0.267	0.223	0.196	0.314	0.000	0.196	2.014	1.495	1.454	1.395	1.248

tumor	hfl-B5	hfl-B5.aAug05	1123	0.225	0.226	0.252	0.256	0.040	0.185	0.897	0.772	0.835	0.654	0.686

tumor	COL3A1	COL3A1.aAug05	1680	0.184	0.363	0.195	0.258	0.000	0.184	0.228	0.336	0.230	0.183	0.168

tumor	DNAJB11	DNAJB11.aAug05	1612	0.265	0.281	0.271	0.183	0.000	0.183	1.094	1.165	1.316	1.280	1.286

*Note: this is the brief version of additional file 6

We were able to validate that all predicted exon junctions were actually observed in the transcriptome when the samples were independently analyzed with qRT-PCR. Six of the 10 candidate exon junctions analyzed with qRT-PCR have a level of expression in the normal lung sample within the range of expression of the 4 MPM tumors. As far as the differential expression was concerned, we required that the individual expression level of each exon junction in at least 2 of the 4 MPM tumors (and the average for all 4 MPM tumors) be in the predicted direction relative to the level in the normal lung sample. We were able to validate the differential expression of 8 of the 10 exon junctions. Differential expression of exon junctions 'EMP2.shed3.aAug05' (over-expressed in normal) and 'DNAJB11.aAug05' (over-expressed in tumor) observed using 454 sequence data could not be confirmed by qRT-PCR. In both cases, expression of the exon junction was detected at similarly high levels in all samples using qRT-PCR (see table 1 and additional file 6 for detail comparison between qRT-PCR results and transcriptome sequencing results).

**Figure 2:**
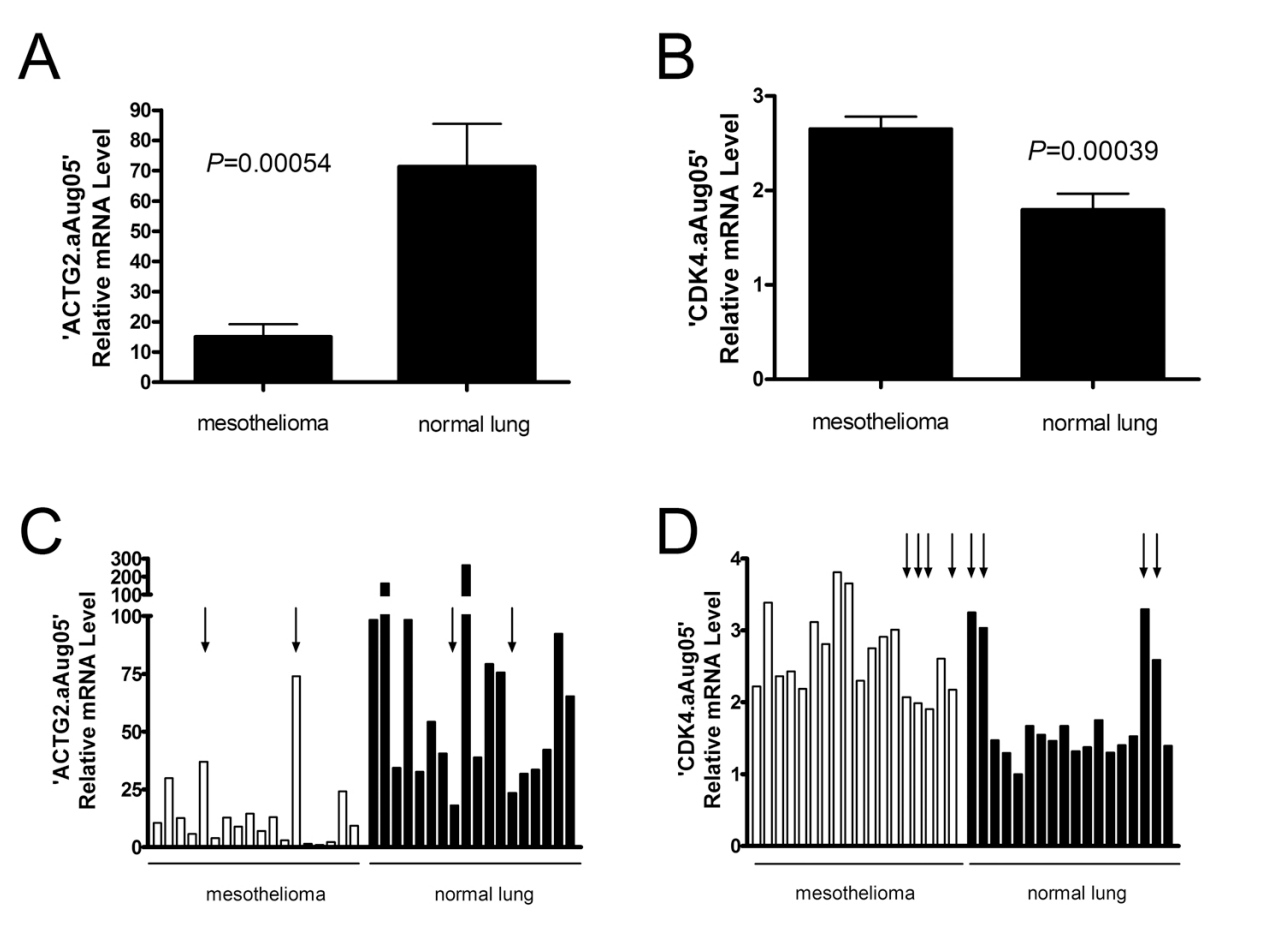
**Differential expression of specific exon junctions in MPM and normal lung samples**. Average expression levels of exon junctions in ACTG2 (A) and CDK4 (B) differ statistically significantly between MPM and normal lung samples. Individual expression levels of exon junctions in ACTG2 (C) and CDK4 (D) can be used to classify MPM and normal tissue samples using the median value as a cut-off. Error bars, SEM. Arrows indicate misclassified samples.

Among the 8 validated differentially expressed exon junctions, we selected 'ACTG2.aAug05' and 'CDK4.aAug05' for further evaluation in a set of 18 additional MPM samples and 18 normal lung samples using qRT-PCR. These two exon junctions were selected because they were associated with the greatest magnitude of differential expression in the predicted direction for each of the 5 discovery samples using both 454 read counts and qRT-PCR. We found that the average expression levels of both 'ACTG2.aAug05' and 'CDK4.aAug05' were statistically significantly different in the predicted direction between normal and tumor samples (*P *= 0.00054 and *P *= 0.00039, respectively, Figure 2A and 2B). 'ACTG2.aAug05' was associated with a greater range of expression levels across individual samples (~100-fold difference) than 'CDK4.aAug05' (~3-fold difference).

Using the median value of relative exon junction expression levels in all 36 samples as a cut-off, we found that 'ACTG2.aAug05' expression correctly classified 89% (16/18 tumor, 16/18 normal) of the samples (*P *= 5.2 × 10^-6^, Figure 2C), whereas 'CDK4.aAug05' correctly classified 78% (14/18 tumor, 14/18 normal) of the samples (*P *= 2.2 × 10^-3^, Figure 2D). All 36 samples were correctly classified by at least one exon junction. Using G*Power, post-hoc power analysis show sufficient sample size with Power (1-β err prob) = 1.000 for ACTG.aAug05 and Power (1-β err prob) = 0.997 for CDK.aAug05.

## Discussion

There have been previous reports of alternatively spliced cancer-associated genes that could serve as diagnostic and prognostic markers as well as guide to potential therapeutic targets [10-12]. These studies used a variety of tools including analysis of publicly available databases and discovery using microarrays, both of which utilize high-throughput methods to characterize cancer related alternative gene splicing [13,20]. More recently, a high throughput RT-PCR method has been used to measure the differential expression of 3,327 alternative splicing events in 600 cancer-related genes for ovarian and breast cancer [3,21]. The present study provides one of the first attempts to show how whole transcriptome sequencing using massive parallel sequencing technology can be used to simultaneously profile as many as 151,486 alternative splicing events in cancer patient samples for all the human genes cataloged in the comprehensive AceView database of transcribed sequences. In our previously study [15], we used the Roche/454 next-generation sequencing system for whole transcriptome pyrosequencing of cDNA samples for 4 MPM tumors, 1 lung adenocarcinoma and 1 normal lung. We generated between 2.5 and 2.9 million shotgun sequencing reads for each sample of average length ~105 bp and focused on the identification of novel Single Nucleotide Polymophisms (SNPs) in the expressed sequences. Herein, we hypothesized that these same transcriptome reads could be used to detect alternatively spliced genes in the patient samples by mapping all the read sequences onto known exon junction sequences as virtual probes. Using a subset of the same dataset [15], we have developed a software pipeline to quantify the expression levels of exon junctions by counting the number of reads that match to each exon junction to identify cancer related alternative splicing pattern.

In this study, we have identified several genes expressing alternatively spliced transcripts at different levels in the tumors and in the matching normal lungs. Many of these genes have been previously implicated in cancer. ACTG2 contains one 5' un-translated exon and 8 coding exons spanning 27 kb [22]. This gene has 7 known splicing variants shown in AceView website [16], but none of them has been directly related to cancer. An expression microarray analysis performed on a derivate breast cancer cell line resistant to cisplatin showed that ACTG2 expression increases in the chemotherapy resistant cell line compared to the normal indicating that it may be associated with cisplatin resistance [23]. In another study, ACTG2 expression was identified as cadmium-responsive [24]. The authors concluded that repressed expression of ACTG2 following cadmium exposure may contribute to the cell cycle arrest. CDK4 is a well-known for its role in cancer [25]. It is a cyclin-dependent kinase-4 involved in the cell cycle and can both start and stop the cell cycle in response to proliferative or anti-proliferative signals. It has 13 known splice variants according to AceView website [16]. Several reports have already linked CDK4 expression to mesothelioma [26,27]. However, this was the first study showing that different CDK4 splice variants have differential expression levels in MPM and matching normal lung.

The differentially expressed splice variants for ACGT2 and CDK4 were specifically chosen for further examination using qRT-PCR in additional 18 MPM and matched normal lung samples. This analysis suggested that the differentially expressed splice variants may provide reliable markers for disease and be used to classify the samples with high sensitivity and specificity.

Several of the other genes that appeared to exhibit differentially expressed splice variants in the present study have also been implicated in cancer and would be worthy of further study. CYFIP1 has been shown to be a novel tyrosine kinase substrate in a breast cancer model [28]. Interestingly, the differentially expressed transcript (CYFIP1.fAug05) is not included in NCBI Refseq sequence database. It has been suggested that COL3A1 could be a potential diagnostic marker for large B-cell lymphoma (DLBCL) as is shows statistically significant different expression between DLBCL and follicular lymphoma [29]. In addition, COL3A1 expression has been related to resistance to platinum drugs in ovarian cancer [30]. TXNRD1 is a key enzyme in the regulation of the intracellular redox environment [31]. Transcription of TXNRD1 involves alternative splicing, leading to a number of transcripts. In particular, expression of the TXNRD1_v3 transcript has been found in several cancer cell lines [32]. Recently, its locus has been associated with advanced colorectal adenoma by epidemiologic and animal studies [33].

In this pilot study, we were not able to find statistical difference between differentially expressed exon junctions because of the small sample size (only 4 MPM and 1 normal lung sample), neither we were able to compare the results among different platforms. In addition, not all exon junctions predicted to be differentially expressed proved to be so to the same extent when examined with the qRT-PCR. This is likely due the limited number of specimens examined. Furthermore, the signal to noise ratio may have been over-amplified as not to miss any potential differentially expressed candidates. After all, the EJEI was designed to magnify the differential expression and is not in the same order of magnitude as the actual expression of the exon junctions. Other potential limitations may be due to sequencing artifacts, insufficient sequencing depth, SNPs near the EJ and incomplete database for possible exon junctions. These limitations may be avoided using other next-generation sequencing platforms, such as Helicos True Single Molecule Sequencing without amplification, Illumina or SOLiD [34], or by increasing the sequencing depth. Nevertheless, at least 2 of the top 10 exon junctions prioritized for analysis remained differentially expressed in most tested specimens supporting the utility of this approach.

The present study provides an example of a possible application of advanced sequencing technologies in cancer research. The current sequencing technologies are now capable of generating millions of shotgun transcriptome reads in a matter of days. For example, the latest Roche/454 GS FLX Titanium system generates over 1,000,000 reads of average length 400 bp in one 10-hour sequencing run providing orders of magnitude improvement in speed and cost over conventional Sanger-based sequencing. One of the great virtues of the shogun transcriptome sequencing process is that there is no need to impose any bias for known genes, exons, or splice-junctions as required for example with exon microarrays. As these technologies continue to improve their throughput and read lengths and lower their costs, they promise to revolutionize gene expression analysis by simultaneously providing information about expression levels, transcript variants, and SNPs.

The greater challenge for the successful application these technologies for our understanding of health and disease will be the analysis and interpretation of the data. Here we have introduced a data analysis pipeline to map 13,274,187 transcriptome reads from patient cDNA samples onto 151,486 known splice junctions cataloged in the comprehensive AveView database of transcribed sequences. However, in short order we can expect that the competing next generation sequencing technologies will be generating several orders of magnitude more transcriptome and genomic sequencing data for a wide variety of human diseases and cancer. Further advances in the bioinformatics analysis of this flood of data are clearly required, for example, to map sequencing reads directly to human genome to identify novel transcribed sequences and genes, alternative exons and splicing events, and possible gene fusions in patient samples

## Conclusion

We demonstrated that whole-transcriptome shotgun sequencing and downstream bioinformatics pipeline can be powerful high-throughput tools for the identification of differentially expressed exon junctions resulting from alternative splicing variants. The alternative transcripts discovered in the study could be useful as diagnostic markers as well as potential therapeutic targets for MPM.

## Competing interests

The authors declare that they have no competing interests.

## Authors' contributions

LD carried out the Perl programming, data analysis and PCR experiments. LD, RJ, GJG, ADR and RB drafted the manuscript. LD, RJ and RB conceived of and designed the study. RB and DJS provided access to the transcriptome dataset and provided critical manuscript review. YX provided computing support for the Partners High Performance Computing Cluster facilities. All authors read and approved the final manuscript.

## Pre-publication history

The pre-publication history for this paper can be accessed here:

http://www.biomedcentral.com/1471-2350/10/149/prepub

## Supplementary Material

Additional file 1 PCR Primers used to quantify expression levels of candidate exon junctions Click here for file

Additional file 2 Methods to calculate exon junction expression index (EJEI) using RT-PCR Click here for file

Additional file 3 Distribution of the 70,953 exon junctions mapped by at least one transcriptome read in at least one patient sample among 5 discovery samples Click here for file

Additional file 4 76 highly expressed exon junction in MPM with EJEI difference > 0.1 and their mapped read numbers, EJEI, normalized EJEI and EJEI difference between MPM samples and normal lung Click here for file

Additional file 5 32 highly expressed exon junction in normal lung with EJEI difference > 0.1 and their mapped read numbers, EJEI, normalized EJEI and EJEI difference between normal lung and MPM samples Click here for file

Additional file 6 Mapping results for the 10 candidate differentially expressed exon junctions, their exon junction expression index (EJEI), EJEI difference between mesothelioma and normal samples and the RT-PCR results Click here for file
