# Supplementary material for: Differentially expressed alternatively spliced genes in Malignant Pleural Mesothelioma identified using massively parallel transcriptome sequencing
Source: BMC Med Genet. 2009 Dec 31;10:149. doi: 10.1186/1471-2350-10-149 (PMC2808307; doi:10.1186/1471-2350-10-149)
Supplement: Additional file 1 — PCR Primers used to quantify expression levels of candidate exon junctions [file 1471-2350-10-149-S1.DOC]

**Additional file 1, PCR Primers used to quantify expression levels of candidate exon junctions**

| **Gene^a^** | **Aceview Exon Junction Identifier^b^** | **NCBI Accession #** | **Forward Primer Sequence**  **(5’-3’)** | **Reverse Primer Sequence**  **(5’-3’)** | **Forward Primer Location (Aceview/NCBI)^c^** | **Reverse Primer Location (Aceview/NCBI)^c^** | **Location of Exon Junction**  **(NCBI)** | **PCR Amplicon Size** |
| --- | --- | --- | --- | --- | --- | --- | --- | --- |
| ACTG2 | ACTG2.aAug05.574 | NM_001615.3 | TTCAATGTCCCTGCCATGTA | GCAGGGCATAGCCTTCATAG | 504/504 | 639/639 | 574 | 136 |
| ACTG2 | ACTG2.aAug05.CS | NM_001615.3 | TTGCTGACAGGATGCAGAAG | TGGAGAGAGAGGCCAGGATA | 1051/1051 | 1176/1176 |  | 126 |
| CDK4 | CDK4.aAug05.1246 | NM_000075.2 | CCCGAAGTTCTTCTGCAGTC | CTGGTCGGCTTCAGAGTTTC | 1160/774 | 1279/893 | 860 | 120 |
| CDK4 | CDK4.aAug05.CS | NM_000075.2 | GGCCCTCAAGAGTGTGAGAG | ACATTGGGATGCTCAAAAGC | 709/323 | 825/439 |  | 117 |
| C1QC | C1QAandC1QG.aAug05.1449 | NM_172369.2 | TCTGCATCCTTGCCTAGACC | AAGTGTCCCAGGAACCACAC | 1422/889 | 1575/1042 | 916 | 154 |
| C1QC | C1QAandC1QG.aAug05.CS | NM_172369.2 | GTGTGGTTCCTGGGACACTT | TATATCTCCCCACCCCTTCC | 1539/1023 | 1629/1113 |  | 91 |
| CYFIP1 | CYFIP1.fAug05.49 | NM_014608.2 | CGCAGAACAGGGGTCCTTA | CTGGTAGAGCAGCGAGGATG | 44/NA | 198/NA | NA | 155 |
| CYFIP1 | CYFIP1.fAug05.CS | NM_014608.2 | GGATGTATTTGACGCCCAGT | GACACTCCCATCCATCAGGT | 836/859 | 915/938 |  | 80 |
| EMP2 | EMP2.shed3.aAug05.1003 | NM_001424.4 | GACCCATCCACCATTCATTC | ATAGAAGGCCAGACGCAGTG | 927/1801 | 1030/3960 | NA | 104**^d^** |
| EMP2 | EMP2.shed3.aAug05.CS | NM_001424.4 | CGTCGGGGCTACATGAGTAT | GATGTGGGCACTGAATGTTG | 1123/4053 | 1223/4153 |  | 101 |
| MRPL51 | MRPL51.bAug05.542 | NM_016497.2 | AGAAATGGCAGGGAACCTCT | TCCAACGATCAACCACTTTG | 459/152 | 604/297 | 235 | 146 |
| MRPL51 | MRPL51.bAug05.CS | NM_016497.2 | CAAAGTGGTTGATCGTTGGA | CGTTGCAATTCATTCCCTTT | 585/278 | 731/424 |  | 147 |
| TXNRD1 | TXNRD1.aAug05.1333 | NM_001093771.1 | GAACAAATTGAAGCAGGGACA | TCTTGTGCAAGCATCTCTTCC | 1243/1241 | 1368/1366 | 1331 | 126 |
| TXNRD1 | TXNRD1.aAug05.CS | NM_001093771.1 | AGCATCCTATGTCGCTTTGG | TGCCATGTTCTTCCATGTGT | 1065/1063 | 1207/1205 |  | 143 |
| hfl-B5 | hfl-B5.aAug05.1123 | NM_006360.3 | TTGACACAATGCAGCAAGAA | TCCGATGTGTGCTATGACTGA | 999/924 | 1139/1064 | 1048 | 141 |
| hfl-B5 | hfl-B5.aAug05.CS | NM_006360.3 | TGATGCCCACAGGTGTATTG | CATGAATAAGCTCGCCTTCC | 724/649 | 833/758 |  | 110 |
| DNAJB11 | DNAJB11.aAug05.1612 | NM_016306.4 | GAGAGACGGCATGGAGTACC | TGGGTGCTTGACAACTTTGA | 1577/868 | 1676/967 | 903 | 100 |
| DNAJB11 | DNAJB11.aAug05.CS | NM_016306.4 | TGCAGGAAATTTTGTGGAAG | AGACCACCTCCTGGGTCATT | 1367/658 | 1506/797 |  | 140 |
| COL3A1 | COL3A1.aAug05.1680 | NM_000090.3 | ATGGCATCCCAGGAGAAAAG | GTTCTCCAGCAGCTCCTCTG | 1660/1607 | 1740/1687 | 1627 | 81 |
| COL3A1 | COL3A1.aAug05.CS | NM_000090.3 | GGTGCTAAGGGTGAAGTTGG | CGCCTTTACCACCAGGACTA | 1212/1159 | 1359/1306 |  | 148 |

^a^NCBI Entrez gene symbol

^b^Nomenclature used to describe exon junctions detailed in Methods. For example, the exon junction ‘ACTG2.aAug05.574’ defines a junction that occurs in the ACTG2 gene in Aceview transcript ‘a’ in the ‘Aug05’ database at nucleotide position 574. CS – Common sequence; used as a reference mRNA sequence for relative quantification of exon junction expression levels. See Methods.

^c^Primer start location along the Aceview mRNA sequence or the NCBI sequence.

NA; Not available. The forward primer and location for this exon junction is contained within an intron in the NCBI database.

^d^Because primers cover big range (2160bp) on NCBI Refseq sequence NM_001424.4, PCR does not generate PCR product due to short extension time of the PCR reaction.
