# Supplementary material for: Differentially expressed alternatively spliced genes in Malignant Pleural Mesothelioma identified using massively parallel transcriptome sequencing
Source: BMC Med Genet. 2009 Dec 31;10:149. doi: 10.1186/1471-2350-10-149 (PMC2808307; doi:10.1186/1471-2350-10-149)
Supplement: Additional file 2 — Methods to calculate exon junction expression index (EJEI) using RT-PCR [file 1471-2350-10-149-S2.DOC]

Additional file 2:

Method to calculate the relative exon junction expression level using Quantitative RT-PCR:

The comparative CT equation (Applied Biosystems) describes the exponential nature of PCR-based amplification and was used to obtain quantitative values for exon junction expression levels. The “CT” term stands for the fractional PCR cycle at which the quantity of the amplified product reaches a pre-determined threshold. The comparative CT equation states that the expression level of a gene in a given sample, normalized within the sample to an endogenous reference gene, and relative to the expression level of the same gene in another sample (i.e. an arbitrarily chosen “calibrator sample”) can be represented as: 2^-ΔΔCT^ where ΔΔCT=[ΔCT_(sample “x”)_]-[ΔCT_(calibrator sample)_] and ΔC_T_=[CT_(target gene)_]-[CT_(reference gene)_]. The “target gene” PCR product was designed to include the relevant exon junction. The “reference gene” PCR product was designed to target a common sequence region among all or most of the AceView transcripts of the same gene.
